# 

**Published:** 1907-07

**Authors:** 


### INDEX.

A Brief Epistle to the Esculapians........................449
Abstracts and Selections.........70, 101, 150, 188, 230, 275, 321, 372, 422, 458........................................516
A Great Reformation Needed: A Call to Duty. By Alexander S. Barrett, M. D., Springtown, Texas....................359
A Medical Mosaic—the Composite Board of Medical Examiners 91
American Proctologic Society............................ 49
Atrophic Cirrhosis of the Liver. By John V. Shoemaker, M. D., LL. D.................................................209

Books and Magazines........23, 108, 158, 200, 288, 338, 378, 431
Breakers Ahead ..........................................267

Cause and Management of Non-Specific Infection of the Extremities. By L. Sexton, M. D., Tulane University......443
Chronic Intestinal Auto-Intoxication. By R. H. L. Bibb, M. D., Saltillo, Mexico................................348
Cold as a Therapeutic and Prophylactic Agent. By R. D. Robinson, M. D., Torreon, Coah., Mexico...................401
Commonsense Scientific Quar[illegible]ntine Methods. By C. W. Trueheart, M. D., Galvest[illegible]n, T[illegible]xas.........................495
Communications........... ...................146, 419, 511

Death of Dr. Law.........................................318
Department of Public Health..............................455
Dr. Coe and the Octopus Management.......................319
Dr. J. Q. Burton's Treatment for Typhoid Fever...........408
Dr. Sajous' Great Work...................................224
Drunkard's Daughter, The................................. 13

Echoes of the Corpus Christi Meeting.....................507
Editorialettes......20, 68, 145, 186, 225, 270, 320, 371, 417, 451

First Fruits of "Unification"............................179
Future Science of Medicine, The. By J. Madison Taylor, A. B., M. D., Philadelphia, Pa..............................213

House Disinfection. By Theo. Y. Hull, B. S., M. D., San Antonio, Texas ...........................................353
Hyperemesis Gravidarum. By W. T. Marrs, M. D., Peoria, Illinois ................................................170

In Darkest Africa........................................317
Interview with State Health Officer Brumby............... 18
Important Ruling: Powers of Boards of Health.............450

Letter from Dr. J. Q. Burton.............................362

Musca Domestica in Tuberculosis. By Theo. Y. Hull, B. S., M. D., San Antonio, Texas................................499

Our Greatest (and Most Neglected) National Asset..........365

Partial Thyroidectomy in the Treatment of Exophthalmic Goitre. By Aime Paul Heineck, Chicago..................299
Politics and the State Medical Association..................223

President's Address, Central Texas District Medical Society, Section on Obstetrics and Gynecology. By W. L. Crosthwaite, M. D., Holland, Texas..........................173
Publisher's Notes...32, 78, 110, 160, 202, 247, 294, 342, 380, 434, 482,..........................................522

Recumbency in the Treatment of Infantile Paralysis. By Adoniram B. Judson, M. D., New York, N. Y................ 47
Rockefeller, the Philanthropist..........................269

Some Pathological Causes of Dysmenorrhea............... 83
Shock in Its Relation to Anesthesia. By Emory Lanphear, M. D., Ph. D., LL. D., St. Louis, Mo..................262
Society Notes ..........................................285
Some Practical Hints on Prostatectomy. By G. Frank Lydston, M. D., Chicago, Illinois..............................409
Social Hysteria. By G. Frank Lydston, M. D..............437

The Cause and Prevention of Rape. By F. E. Daniel, M. D., Austin, Texas .......................................391
The Moloch of Civilized Man............................ 63
The Moralities of Medicine. By Charles M. Rosser, M. D., Dallas, Texas .......................................165
The Texas Health Officers' Association. Secretary's Report...485
The Treatment of Extra-Uterine Pregnancy. By Aime Paul Heineck, Chicago, Ill................................ 1
Treatment of Infected Wounds. By Emory Lanphear, M. D., Ph. D., LL. D., St. Louis, Mo.......................... 43
The Treatment of Pneumonia. By W. T. Marrs, M. D., Peoria, Illinois ................................................171
Tuberculosis Problem in Texas.......................... 15

Urinary Ailments: Therapeutics. By W. C. Abbott, M. D., Chicago, Illinois.....................................176

Very Important Notice...................................413

Wright's Principles of Vaccine Therapy. By H. N. Graves, M. D., Georgetown, Texas............................. 255

Yellow Fever Again in Cuba. By A. M. Fernandez De Ybarra, A. B., M. D., New York City..........................123

X-Ray as an Aid in the Early Diagnosis of Pulmonary Tuberculosis, The. By R. H. L. Bibb, M. D., Saltillo, Mexico....345

